# Evolutionary factors affecting *Lactate dehydrogenase *A and B variation in the *Daphnia pulex *species complex

**DOI:** 10.1186/1471-2148-11-212

**Published:** 2011-07-18

**Authors:** Teresa J Crease, Robin Floyd, Melania E Cristescu, David Innes

**Affiliations:** 1 Department of Integrative Biology, University of Guelph, Guelph, Ontario N1G 2W1, Canada; 2 Great Lakes Institute for Environmental Research, University of Windsor, Windsor, Ontario N9B 3P4, Canada; 3 Department of Biology, Memorial University of Newfoundland, St. John's, Newfoundland and Labrador A1B 3X9, Canada; 4 School of Clinical Sciences, Southmead Hospital, University of Bristol, Bristol BS105NB, UK

## Abstract

**Background:**

Evidence for historical, demographic and selective factors affecting enzyme evolution can be obtained by examining nucleotide sequence variation in candidate genes such as *Lactate dehydrogenase *(*Ldh*). Two closely related *Daphnia *species can be distinguished by their electrophoretic *Ldh *genotype and habitat. *Daphnia pulex *populations are fixed for the S allele and inhabit temporary ponds, while *D. pulicaria *populations are fixed for the F allele and inhabit large stratified lakes. One locus is detected in most allozyme surveys, but genome sequencing has revealed two genes, *Ldh*A and *Ldh*B.

**Results:**

We sequenced both *Ldh *genes from 70 isolates of these two species from North America to determine if the association between *Ldh *genotype and habitat shows evidence for selection, and to elucidate the evolutionary history of the two genes. We found that alleles in the pond-dwelling *D. pulex *and in the lake-dwelling *D. pulicaria *form distinct groups at both loci, and the substitution of Glutamine (S) for Glutamic acid (F) at amino acid 229 likely causes the electrophoretic mobility shift in the LDHA protein. Nucleotide diversity in both *Ldh *genes is much lower in *D. pulicaria *than in *D. pulex*. Moreover, the lack of spatial structuring of the variation in both genes over a wide geographic area is consistent with a recent demographic expansion of lake populations. Neutrality tests indicate that both genes are under purifying selection, but the intensity is much stronger on *Ldh*A.

**Conclusions:**

Although lake-dwelling *D. pulicaria *hybridizes with the other lineages in the pulex species complex, it remains distinct ecologically and genetically. This ecological divergence, coupled with the intensity of purifying selection on *Ldh*A and the strong association between its genotype and habitat, suggests that experimental studies would be useful to determine if variation in molecular function provides evidence that LDHA variants are adaptive.

## Background

Understanding the evolutionary factors that affect genetic variation in natural populations remains the primary focus of population genetics. Early surveys of allozyme variation using protein electrophoresis revealed abundant polymorphism, but only about one third of the possible amino acid changes, and none of the variation at silent and non-coding sites, could be detected with this technique [1]. Analyses of genetic variation now involve nucleotide data, which can be used in a variety of statistical tests to discriminate between patterns of variation due to neutral processes (drift, population expansion, bottlenecks) and various types of selection (positive, purifying, balancing). Most studies have shown that levels of variation at silent and non-coding sites are substantially higher than levels of protein variation, suggesting that the majority of non-synonymous mutations are deleterious [1]. Even so, patterns of allozyme (protein) variation often appear to conform to neutral or nearly neutral expectations [2]. Conversely, detailed studies showing associations with different habitats, or clinal variation across wide geographic ranges have provided evidence that functional differences among allozyme variants can affect fitness (e.g. Alcohol dehydrogenase and Glucose-6-phosphate dehydrogenase in *Drosophila melanogaster *and Phosphoglucose isomerase in *Colias *butterflies; reviewed in [3]). Thus, a major challenge has been to determine the adaptive significance, if any, of polymorphism and interspecific divergence at allozyme loci [4].

L-Lactate dehydrogenase (L-LDH, EC 1.1.1.27) is involved in the interconversion of pyruvate and L(-)-lactate, which allows the aerobic metabolism of lactate accumulated by anaerobic glycolysis following periods of exposure to lowered environmental oxygen tension, or hypoxia [5]. There is evidence that selection is acting on LDH variation in well-studied species such as the killifish, *Fundulus heteroclitus *[6,7]. For example, an amino acid substitution between two alleles that vary along a cline in temperature affects thermostability [8], and experimental studies have shown that this variation is involved in the adaptation of killifish to hypoxia [9,10].

A strong association between *Ldh *genotype and habitat has been also observed in members of the *Daphnia pulex *species complex. Based on relative electrophoretic mobility, the pond species, *D. pulex *is characterized by homozygosity for the slow (S) *Ldh *allele and the closely related lake species, *D. pulicaria *by homozygosity for the fast (F) allele in North America [11-13]. In addition, the two species hybridize and hybrids are generally found in ponds or disturbed, intermediate habitats [13]. The genetic and habitat segregation is also associated with differences in physiological and life history traits [14,15]. Surveys of allozyme variation in most *Daphnia *species are consistent with a one-locus model, but a recent analysis of the *D. pulex *genome sequence has identified two *Ldh *genes, *Ldh*A and *Ldh*B, that are approximately 26 cM apart [16,17]. Preliminary gene expression work suggests that *Ldh*A is expressed at a significantly higher level than *Ldh*B [18], but the link between the allozyme locus and the genome sequences has not yet been made. Nevertheless, the fixation of different LDH variants in *D. pulex *and *D. pulicaria *in different aquatic habitats, despite the fact that they share polymorphisms at other allozyme loci [19-21], suggests the possibility that *Ldh *variation is adaptive. However, the hypothesis that selection has played a role in *Ldh *divergence in *Daphnia *has yet to be tested.

Genetic studies suggest that *D. pulex *and *D. pulicaria *are members of a relatively young species complex and appear to be undergoing ecological speciation [22]. Both species reproduce by cyclical parthenogenesis in which females produce offspring by apomictic parthenogenesis during favorable conditions and have a sexual phase that results in the production of diapausing eggs prior to the onset of unfavorable conditions. Natural hybrids between the two species reproduce by obligate parthenogenesis [23,24] in which females produce broods of parthenogenetic offspring (like cyclic females), but also produce their diapausing eggs parthenogenetically. This results in a completely ameiotic life history and allows clonal genotypes to persist for many years.

The objectives of the present study are to analyze both *Ldh*A and *Ldh*B sequence variation to (1) determine which locus is detected in allozyme surveys, (2) elucidate the evolutionary history of the two genes in the context of speciation and hybridization, and (3) determine if there is evidence that selection is acting on the variation at each locus. To achieve these objectives, isolates of *D. pulex *and *D. pulicaria *from North America were chosen to maximize geographic coverage of the three *Ldh *genotypes (SS, SF and FF) and the two breeding systems.

## Methods

### *Daphnia *samples

**Figure 1:**
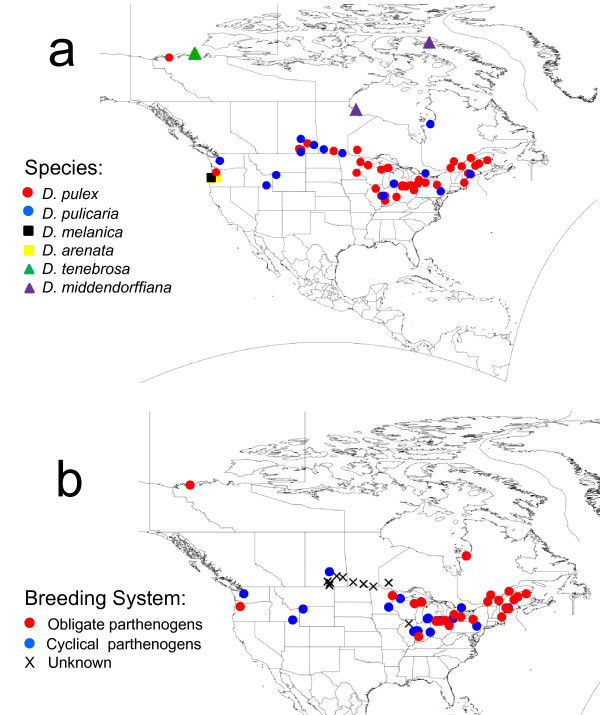
**Location of the North American *Daphnia *populations sampled for this study**. Latitude and longitude for each site are available in Additional file 1.1. Populations are characterized by **(a) **species and **(b) **breeding system for *D. pulex *and *D. pulicaria *isolates.

**Figure 2:**
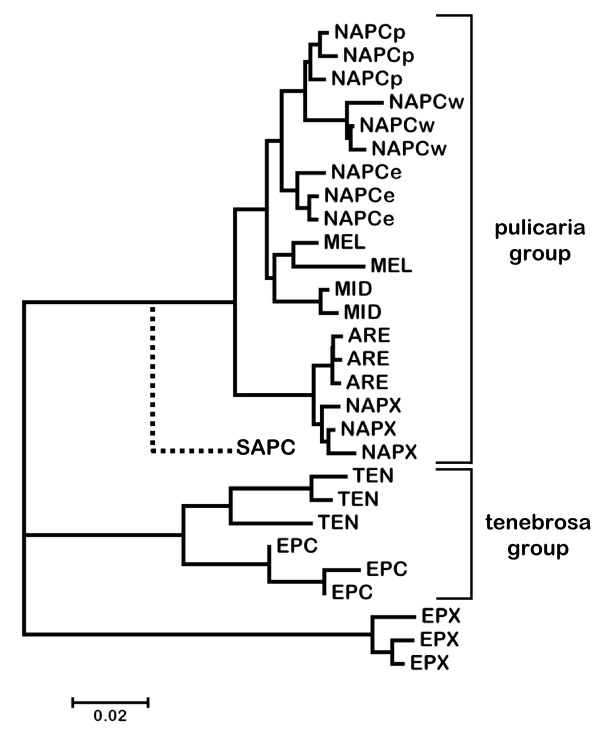
**Neighbor-joining tree of lineages in the *Daphnia pulex *species complex**. Representative sequences of the mitochondrial *ND*5 gene were taken from Colbourne *et al. *[25]. The relationship of the SAPC-C lineage, first identified by Mergeay *et al. *[26], to the rest of the complex is based on the mitochondrial *12S rRNA *gene. Attempts to amplify the *ND*5 fragment from members of this lineage have been unsuccessful. Taxa are indicated as follows: ARE = *D. arenata*, EPC = European *D. pulicaria*, EPX = European *D. pulex*, MEL = *D. melanica*, MID = *D. middendorffiana*, NAPC = North American *D. pulicaria *(e = eastern, w = western, p = polar), NAPX = North American *D. pulex*, SAPC = South America *D. pulicaria*, TEN = *D. tenebrosa.*

A total of 85 *Daphnia *isolates collected from 68 populations, mainly from North America, were included in this study (Figure 1, Additional file 1.1). These isolates include representatives of all the major lineages in the *D. pulex *species complex identified by Colbourne et al. [25] using phylogenetic analysis of mitochondrial DNA (Figure 2), as well as the C lineage of South American *D. pulicaria *identified by Mergeay et al. [26]. An isolate of *Daphnia obtusa *from Illinois [27] was also included as an outgroup for phylogenetic analyses.

Genomic DNA was generally extracted from multiple parthenogenetically-produced offspring of a single progenitor female using the CTAB method [28]. Isolates from South America and Europe were extracted from single individuals preserved in 95% ethanol using the Mammalian genomic DNA GenElute kit (Sigma-Aldrich).

The breeding system (cyclic or obligate parthenogenesis) of most lab-reared isolates was diagnosed by examination of diapausing egg cases (ephippia) produced in the absence of males. While cyclical parthenogens often release empty ephippial cases unless the eggs have been fertilized, obligate parthenogens deposit eggs into ephippia even in the absence of males [29].

The *Ldh *genotype of all of the isolates from North America was determined by allozyme electrophoresis. In most cases, isolates were assigned to a species within the pulex complex [25] based on the sequence of the mitochondrial *NAD dehydrogenase subunit *5 (*ND*5) gene generated by the lab from which the genomic DNA was obtained (Additional file 1.1). However, isolates that were collected from lakes and reproduce by cyclic parthenogenesis (i.e. sexually) were designated as *D. pulicaria *[13,19] even though some of them have a *D. pulex *mitochondrial haplotype. Previous studies have shown that populations of *D. pulicaria *with either mitochondrial type are not distinguishable from one another based on nuclear allozyme and microsatellite variation, but are divergent from cyclically parthenogenetic populations of *D. pulex *[22,30]. Of the 85 isolates included in this study, 70 were classified as either North American *D. pulex *or *D. pulicaria.*

### Polymerase chain reaction, cloning and sequencing

PCR amplification of the two *Ldh *loci from *Daphnia *genomic DNA was carried out using two sets of primers: LDHA-u4F (5'-GAAAATGGCCACCAGCGTCG) and LDHA-1304R (5'-TTGAACTTCATTCAAAGTGGCAGC) for *Ldh*A, and LDHB-u7F (5'-AATCAGAATGCAGACAAAGGCCTC) and LDHB-1477R (5'-TCAGAACACCAAGTTTGACTGAACTTC) for *Ldh*B. Each 25 μl PCR reaction contained 1X Phusion HF buffer, 2 mM MgCl_2_, 2 mmol of each dNTP, 2 pmol of each primer, and 0.5 units of Phusion High Fidelity polymerase (Finnzymes), with 2 μl (20 to 50 ng) of the target genomic DNA. The thermocycling program for the *Ldh*A primers was: 94°C for 2 min; 25 cycles of 94°C for 30 s, 54°C for 30 s, 72°C for 90 s; a further 10 cycles as above except that the 72°C extension time was increased to 2 min; and finally 72°C for 5 min. A similar program was used for the *Ldh*B primers but with an annealing temperature of 50°C.

Amplicons of the expected size (~1.3 kb for *Ldh*A, ~1.4 kb for *Ldh*B) were cloned using the StrataClone Blunt PCR Cloning Kit (Stratagene) following the manufacturer's protocol. Colonies were grown on Luria Broth (LB) agar containing ampicillin (100 μg/ml) and X-gal (20 μg/ml). Glycerol stocks (LB + amp in 30% glycerol) were generated for 24 white colonies from each experiment and archived at -80°C.

Initially, eight plasmids of the cloned PCR product from each isolate were selected for sequencing. The cloned insert was amplified directly from bacterial cells by PCR using the M13F (5'-GTTGTAAAACGACGGCCAGTG) and M13R (5'-CAGGAAACAGCTATGACCATG) sequencing primers, and Platinum *Taq *polymerase (Invitrogen). If an amplicon of the expected size was not obtained from all eight colonies, the process was repeated until at least eight amplicons of the expected size were obtained. Cycle-sequencing of these amplicons in both directions was carried out using the BigDye 3.1 kit (Applied Biosystems) and the M13F and M13R primers. Each 10 μl sequencing reaction contained 0.25 μl of BigDye mix, 1 μl of sequencing buffer, 10 pmol of primer, 6.75 μl of ultrapure water, and 1 μl of PCR amplicon. Completed reactions were run on an ABI3730 DNA Analyzer.

### Sequence diversity analyses

A reference sequence was created from the *D. pulex *genome sequence [18] for each of the *Ldh *genes indicating the location of exons, introns and the start and stop codons. Sequence electropherograms from each cloning experiment were aligned and edited in Sequencher v.4.5 (Gene Codes) using the reference sequences to orient the alignment and to trim the ends to the start and stop codons. The consensus sequence of each allele present within an isolate was assembled into a master alignment of all the *Ldh *alleles for a particular gene. Unique differences among the eight clones from an isolate were assumed to be PCR, cloning or sequencing errors and were excluded from the analysis.

An alignment containing alleles from all isolates was generated for each gene in BioEdit [31]. Separate alignments for the exon and intron sequences were also generated using the reference sequences as a guide.

Analyses of nucleotide diversity included only alleles obtained from isolates of North American *D. pulex *and *D. pulicaria *and their hybrids. Alleles at both loci were divided into pulex and pulicaria groups based on their phylogenetic relationship to alleles obtained from cyclically parthenogenetic isolates from lakes (*D. pulicaria*) and ponds (*D. pulex*). Alleles from isolates that are likely to be hybrids with a species other than North American *D. pulex *or *D. pulicaria *(one isolate of *D. middendorffiana*, three isolates of South American *D. pulicaria*) were excluded from the diversity analyses.

Standard measures of nucleotide diversity including the number of haplotypes (K), the number of segregating sites (S), haplotype diversity (H), the average number of pairwise differences per site between sequences (π) and diversity per site based on the number of segregating sites (θ) were calculated using the program DNASPv5.10 [32]. Both π and θ were estimated for all sites (π_T_, θ_T_), as well as introns (π_i_, θ_i_), synonymous coding sites (π_s_, θ_s_), and nonsynonymous coding sites (π_n_, θ_n_). In addition, DNASP was used to perform tests of neutrality (Tajima's D; Fu and Li's D and D*; Fu and Li's F and F*, Fay and Wu's H). Patterns of diversity between the two loci in each of the pulex and pulicaria allele groups were analysed using an HKA test [33] in DNASP using the *D. obtusa *sequence as an outgroup. We used the algorithm of Betrán et al. [34] in DNASP to detect gene conversion between the pulex and pulicaria allele groups in each locus.

Separate genetic distance matrices were calculated for each allele group (pulex and pulicaria) and each locus based on the number of nucleotide differences between pairs of alleles in Arlequin 3.11 [35]. The geographic distance matrix was constructed by calculating the great circle distance between each pair of sites based on the decimal degrees of latitude and longitude using the function "rdist.earth" with earth radius = 6371 km in the Fields 5.02 package in R 2.9.2 [36]. A Mantel test with 1000 permutations was then conducted between the genetic and geographic distance matrices for each of the four allele groups using Arlequin.

### Phylogenetic analysis

We performed Bayesian phylogenetic analyses on the unique nt sequences (total and introns only, indels excluded) of each *Ldh *gene using Mr. Bayes v3.1.2 [37] with default prior settings and a GTR model. All trees were rooted through a sequence from *D. obtusa*, which is not a member of the pulex complex but a member of its sister species complex [38]. We ran two independent and simultaneous Markov Chain Monte Carlo (MCMC) analyses of 15 heated and one cold chain for six million generations sampling from the chain every 100 generations. The initial 30% of trees were discarded as a burn-in. A 50% majority-rule consensus topology with posterior probability (PP) values for each node was constructed from the post-burn-in trees. We also constructed Neighbor-joining trees [39] of the amino acid sequences using MEGA4 [40] and Poisson-corrected estimates of sequence divergence.

## Results

### Characterization of sequence variation

We obtained a total of 143 *Ldh*A sequences from the 85 isolates included in the study, of which 76 (53.1%) are unique. Our sequences begin at the start codon and end 15 nt upstream of the stop codon. This gene consists of six exons and five introns [18]. The total length of the sequence alignment is 1394 nt with individual sequences varying from 1299 to 1352 nt and all length variation occurring in introns. The coding region is 981 nt resulting in 327 codons for a protein length of 332 amino acids when the five missing from our PCR amplicon are included. The total length of intron sequences in *Ldh*A varies from 318 to 371 nt. The amino acid substitution of Glutamine (uncharged) to Glutamic acid (negative) at amino acid 229 changes the overall charge of the protein, which would cause an electrophoretic mobility shift. Thus *Ldh*A is most likely the locus that is scored in allozyme analyses. The "C" to "G" transversion (CAA to GAA) that causes this substitution corresponds to nt 912 in the alignment (nt 685 in the coding sequence).

We obtained a total of 157 sequences for *Ldh*B of which 115 (73.2%) are unique. This gene consists of eight exons and seven introns [18]. Our sequences begin with the start codon and end with the stop codon resulting in a total alignment length of 1545 nt with individual sequences varying from 1446 nt to 1488 nt. With the exception of a 2-nt insertion in exon 8 of a single sequence, all other length variation occurs in introns. The coding region of 972 nt encodes a protein of 324 amino acids. The total length of intron sequences varies from 471 to 513 nt. The alignments of all the *Ldh*A and *Ldh*B nucleotide and protein sequences are provided as supplementary information (Additional files 2, 3, 4, 5). All unique nucleotide sequences have been submitted to GenBank (accession numbers JN117725-JN117915).

### Phylogenetic analysis

**Figure 3:**
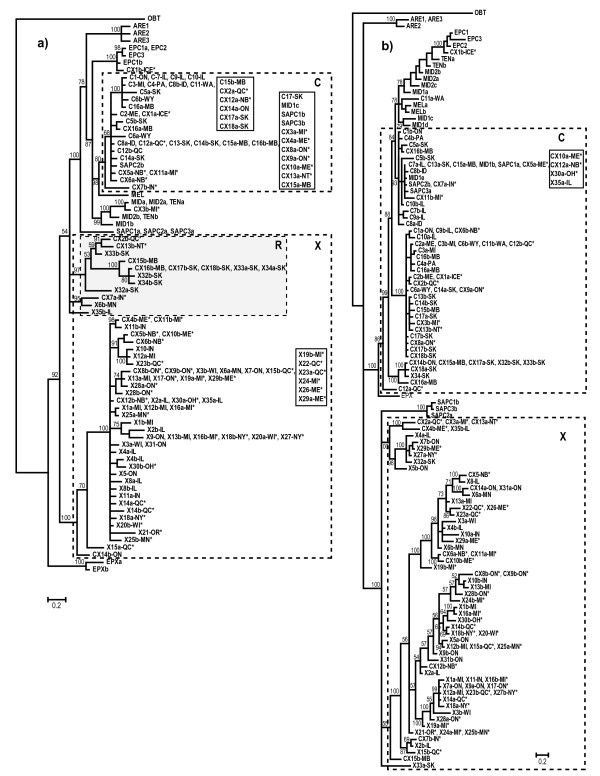
**Bayesian phylogeny of (a) *Ldh*A and (b) *Ldh*B gene sequences from 85 isolates in the *Daphnia pulex *species complex**. The trees are rooted through *Daphnia obtusa*. Species codes are given in Figure 2 except that North American *D. pulicaria *isolates have been shortened to "C" and North American *D. pulex *isolates have been shortened to "X". Hybrids between these two species are designated "CX". Isolates within groups are numbered and alleles within heterozygotes are designated "a" and "b". The 2-letter province or state code is appended to C, X and CX isolates. Allele groups in *D. pulicaria *(C) and *D. pulex *(X) that were used in the nucleotide diversity analyses are boxed. The R box indicates recombinant *Ldh*A alleles (see text). Isolates that reproduce by obligate parthenogenesis are indicated with asterisks (*)

The Bayesian phylogenetic analysis based on complete, unique *Ldh*A sequences shows that all of the fast *Ldh*A alleles from North American (NA) *D. pulicaria *(Additional file 1.2) and hybrids involving this species are monophyletic (Figure 3a). In addition, alleles from NA *D. pulex *form a monophyletic group with the exception of 15 recombinant alleles (described below). Because one allele from this group was isolated from a cyclically parthenogenetic pond population, these alleles were assigned to the pulex allele group. The two alleles from the European *D. pulex *isolate are the sister group to all the other members of the pulex species complex (Figure 3a), which is concordant with the phylogeny based on mitochondrial (mt)DNA ([25], Figure 2). However, the sequences from all the other lineages in the complex cluster with the NA *D. pulicaria *alleles, which is not consistent with the mtDNA phylogeny. For example, *D. tenebrosa *and European *D. pulicaria *form a separate cluster (the tenebrosa group) from species in the pulicaria group in the mtDNA phylogeny (Figure 2). The tree based on *Ldh*A intron sequences (Additional file 6a) is similar to the full-sequence tree but relationships within the cluster of alleles from NA *D. pulicaria*, which also contains alleles from all the other species, are not well resolved.

As is the case for *Ldh*A, *Ldh*B alleles from NA *D. pulex *and *D. pulicaria *form two distinct groups (Figure 3b, Additional file 1.2). In contrast to the *Ldh*A tree, *Ldh*B alleles from *D. arenata *do not belong to either of these major groups and their relationship with them is unresolved. In addition, European *D. pulex *alleles are not the sister group to alleles from all the other species, as is the case for the *Ldh*A and mtDNA trees. The topology based on intron sequences (Additional file 6b) is similar except that the *D. arenata *sequences are the sister group to alleles from NA *D. pulex *and all other sequences cluster with the alleles from NA *D. pulicaria*.

### Recombination between pulex and pulicaria alleles

Analysis of recombination identified gene conversion tracts between the pulex and pulicaria alleles in both genes (Additional file 1.3, 1.4). In most cases these are short (<100 nt) but there are two larger tracts (127 nt in *Ldh*A and 501 nt in *Ldh*B) which both occur in only one allele. With one exception for each gene, conversion tracts consist of pulicaria variants in pulex alleles. A pair of tracts in *Ldh*A; 28 nt from position 812-839 and 74 nt from position1161-1280 (indels are excluded from the tracts), occur in 15 pulex alleles from isolates as far east as Quebec and as far west as the Northwest Territories with most of the alleles occurring in isolates from Manitoba and Saskatchewan. Indeed, these 15 alleles form a cluster that groups more closely with the pulicaria than the pulex *Ldh*A alleles in the phylogenetic tree (Figure 3a).

As most of the *Ldh*A heterozygotes are obligately parthenogenetic (Additional file 1.1), and are thought to be F_1 _hybrids [11,19], we would expect them to be heterozygous for pulex and pulicaria alleles at *Ldh*B as well. This is indeed the case with nine exceptions, six of which come from Manitoba and Saskatchewan (Table 1). In all but one case, the isolate is heterozygous at one locus but homozygous at the other. The exception is an isolate from Saskatchewan that is homozygous for a pulex allele at *Ldh*A but homozygous for a pulicaria allele at *Ldh*B. There is no bias with respect to locus or species in the eight isolates; three are homozygous for the pulicaria allele at one locus, while five are homozygous for the pulex allele. Similarly, *Ldh*A is the homozygous locus in four isolates, while *Ldh*B is the homozygous locus in the other four.

**Table 1:** Potential backcross or F_2 _genotypes of *Ldh*A and *Ldh*B in isolates of *Daphnia pulex *(X), *Daphnia pulicaria *(C) and their hybrids (CX).

	**Genotype**^**2**^	
**Isolate **^**1**^	***Ldh*A**	***Ldh*B**

CX16-MB	CX	CC
CX17-SK	CX	CC
CX18-SK	CX	CC
CX05-NB	CX	XX
X34-SK	XX	CC
X32-SK	XX	CX
X33-SK	XX	CX
X30-OH	XX	CX
X35-IL	XX	CX

1. The last 2 letters are the state or province from which the isolate was collected.

2. C and X refer to allele groups from *D. pulicaria *and *D. pulex*, respectively (Figure 3).

### Amino acid variation

**Figure 4:**
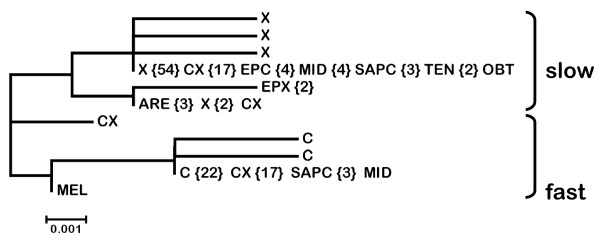
**Neighbor-joining tree of LDHA protein sequences from 85 isolates in the *Daphnia pulex *species complex**. The number of sequences shared by multiple isolates of a particular species is given in brackets. "Slow" and "Fast" refer to electrophoretic mobility in allozyme analyses. C = North American *D. pulicaria*, X = North American *D. pulex *and CX = hybrid isolates. Other taxon codes are defined in Figure 3.

Amino acid variation in LDHA is very limited. Indeed, the outgroup, *D. obtusa*, and most of the lineages in the pulex complex have the same amino acid sequence, which is slow electrophoretically. Fast alleles are only found in *D. melanica*, NA *D. pulicaria *and hybrids, which also include the three SA *D. pulicaria *isolates and one isolate of *D. middendorfficana*. Overall, the 143 nucleotide sequences encode 11 amino acid sequences (Figure 4). Of these, seven are unique and four ("slow", "fast", *D. arenata *and European *D. pulex*) occur more than once. There are nine (2.8%) variable amino acid positions and of these, five variants (55.5%) only occur in one sequence.

The primary differences between pulex (slow) and pulicaria (fast) LDHA are amino acid substitutions at two sites; the substitution of Aspartic acid in pulex for Glutamic acid in pulicaria at position 6, and the substitution of Glutamine in pulex for Glutamic acid in pulicaria at position 229, which likely causes the slow/fast mobility shift. In addition, the *D. arenata *and European *D. pulex *proteins have Asparagine at position 314 while all others have Serine. Finally, the protein encoded by both European *D. pulex *alleles has Isoleucine at position 132 while all other proteins have Leucine.

**Figure 5:**
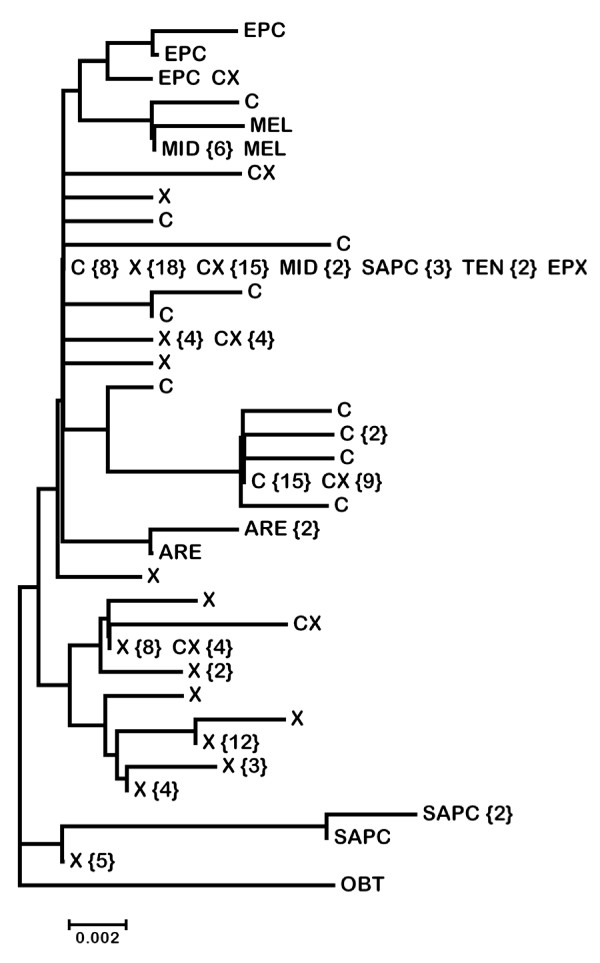
**Neighbor-joining tree of LDHB protein sequences from 85 isolates in the *Daphnia pulex *species complex**. The number of sequences shared by multiple isolates of a particular species is given in brackets. C = North American *D. pulicaria*, X = North American *D. pulex *and CX = hybrid isolates. Other taxon codes are defined in Figure 3.

There is considerably more amino acid variation among LDHB proteins, with little differentiation in any particular lineage (Figure 5). The 157 nucleotide sequences encode a total of 37 protein sequences of which only 14 (37.8%) occur more than once. There are 36 (11.1%) variable amino acid positions and of these, 17 variants (47.2%) only occur in one sequence.

### Sequence diversity and neutrality tests of pulex and pulicaria alleles

**Figure 6:**
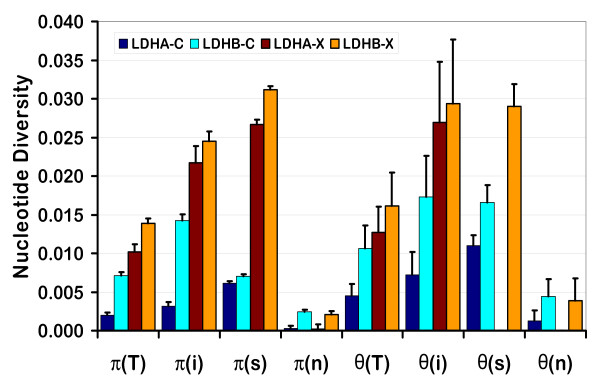
**Nucleotide diversity in *Ldh*A and *Ldh*B alleles from *Daphnia pulex *and *Daphnia pulicaria***. The thin vertical lines represent 1 standard deviation. T = total variation, i = variation in introns, s = variation at synonymous sites in exons and n = variation at non-synonymous sites in exons.

In general, nucleotide diversity (π and θ) is substantially higher in alleles from NA *D. pulex *(pulex alleles) than in alleles from NA *D. pulicaria *(pulicaria alleles) for both genes, and higher in *Ldh*B than *Ldh*A, with the exception of π_s _in pulicaria alleles and π_i _and θ_i _in pulex alleles (Table 2, Figure 6). In addition, diversity tends to be higher at synonymous sites than in introns, except for pulicaria *Ldh*B alleles where diversity is higher in the introns. As expected, nucleotide diversity at nonsynonymous sites is much lower than that at synonymous sites and introns (Table 2). The dN/dS ratios for all four groups of alleles are less than one (Table 3). Pulex *Ldh*A alleles have the lowest dN/dS ratio (0.032) while pulicaria *Ldh*B alleles have the highest ratio (0.348).

**Table 2:** Nucleotide diversity statistics for *Ldh*A and *Ldh*B sequences from the *Daphnia pulicaria *(C) and *Daphnia pulex *(X) allele groups.

**Allele Group **^**1**^	**Total length **^**2**^	**Exon length **^**2**^	**Intron length **^**2**^	**N **^**3**^	**K **^**4**^	**K**_**ex**_^**4**^	**K**_**aa**_^**4**^	**H **^**5**^	**S **^**6**^	**π**_**T **_^**7**^	**π**_**n**_^**7**^	**π**_**s **_^**7**^	**π**_**i **_^**7**^	**θ**_**T **_^**8**^	**θ**_**n **_^**8**^	**θ**_**s **_^**8**^	**θ**_**i **_^**8**^
*Ldh*A-X^S^	1394	981	413	75	44	32	5	0.961	147	0.0103	0.0002	0.0267	0.0217	0.0126	na^9^	na^9^	0.0269
*Ldh*A-C^F^	1394	981	413	42	15	8	4	0.833	29	0.002	0.0003	0.0061	0.0031	0.0045	0.0013	0.011	0.0072
*Ldh*B-X	1545	972	573	71	53	49	16	0.99	199	0.0139	0.0021	0.0312	0.0245	0.0161	0.0039	0.029	0.0294
*Ldh*B-C	1545	972	573	59	39	23	13	0.968	105	0.0071	0.0024	0.007	0.0142	0.0106	0.0044	0.0166	0.0173

1. S and F refer to slow and fast electrophoretic mobility, respectively

2. length in nucleotides based on the total alignment for each gene

3. N = the number of sequences in the allele group, including duplicates. This group of sequences was used to estimate all diversity parameters.

4. K = the number of unique sequences with indels excluded. ex = unique exon sequences, aa = unique amino acid sequences

5. H = haplotype diversity calculated from N sequences with indels excluded

6. S = the number of segregating sites calculated from N sequences with indels excluded

7. π = nucleotide diversity calculated from N sequences with pairwise deletion of indels and Jukes-Cantor correction. T = total sites, n = non-synonymous sites, s = synonymous sites, i = intron sites

8. θ = number of segregating sites calculated from N sequences with pairwise deletion of indels and Jukes-Cantor correction. T = total sites, n = non-synonymous sites, s = synonymous sites, i = intron sites

9. na = not applicable. θ is not estimated when codons differ by multiple changes

**Table 3:** Results of neutrality tests for *Ldh*A and *Ldh*B sequences from the *Daphnia pulicaria *(C) and *Daphnia pulex *(X) allele groups.

**Allele Group **^**1**^	dN/dS	**Tajima D **^**2**^	Fu + Li D* ^**2**^	Fu + Li F* ^**2**^	**Fu + Li D **^**3**^	**Fu + Li F **^**3**^	**Fay + Wu H **^**3**^	**FW- H**^**3 **^**normal**^**4**^
LDHA-X^S^	0.032	-0.31	-2.10	-1.77	-2.04	-1.64	-8.714	-1.010
LDHA-C^F^	0.166	-1.85*	-4.11**	-3.96**	-3.89**	-3.78**	-2.370	-0.655
LDHB-X	0.106	-0.44	-1.64	-1.40	-1.74	-1.40	-7.000	-0.559
LDHB-C	0.348	-1.15	-3.66**	-3.24*	-3.70**	-3.20**	-4.000	-0.471

* = p < 0.05, ** = p < 0.02

1. S and F refer to slow and fast electrophoretic mobility, respectively

2. calculated using all segregating sites

3. calculated using all segregating sites with *D. obtusa *as an outgroup

4. Fay and Wu's H statistic normalized according to Zeng et al. [52]

**Figure 7:**
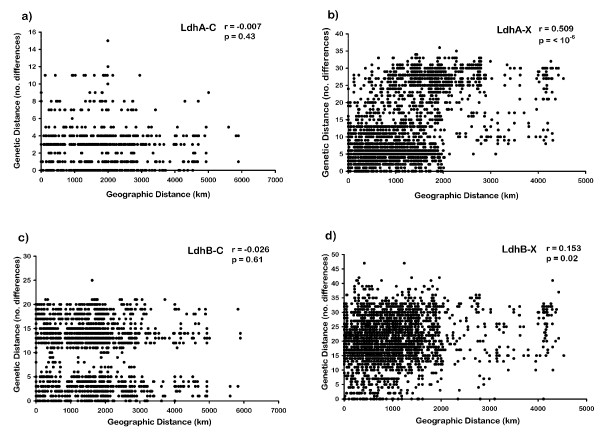
**Mantel tests of the association between geographic distance and nucleotide diversity in *Ldh *sequences from *Daphnia pulex *(X) and *Daphnia pulicaria *(C)**. **(a, b) ***Ldh*A. **(c, d) ***Ldh*B.

The results of the Mantel test indicate that there is no significant correlation between sequence divergence and geographic distance for the pulicaria alleles at either locus (Figure 7a and 7c), but there is a significant positive correlation for the pulex alleles at both loci (Figure 7b and 7d).

All the neutrality tests, except for Fay and Wu's H, are significant for pulicaria *Ldh*A alleles, and the Fu and Li tests are also significant for pulicaria *Ldh*B alleles (Table 2). These test statistics are all negative indicating an excess of rare polymorphisms. The negative values of Fay and Wu's H tests indicate an excess of high frequency variants, but they are not significant. The HKA tests are not significant for either the pulex or pulicaria allele groups (Chi-square (pulex) = 0.215, p = 0.64; Chi-square (pulicaria) = 2.26, p = 0.13), indicating that the ratio of intraspecific polymorphism to interspecific divergence is not significantly different between the two loci.

## Discussion

Analysis of sequence variation in protein coding genes can be used to determine the selective and non-selective processes that have contributed to current patterns of genetic diversity. It has been shown that *Ldh *variation is associated with differentiation between two closely related *Daphnia *species that occupy contrasting habitats, and may be a consequence of ecological speciation. In this study, we found that patterns of variation at two *Ldh *loci are likely a consequence of recent demographic expansion and purifying selection in *D. pulicaria*.

### Phylogeny

It is clear that alleles in lake-dwelling NA *D. pulicaria *and pond-dwelling NA *D. pulex *form distinct groups at both *Ldh *loci. Moreover, alleles from *D. pulex *are divergent from alleles from every other lineage in the pulex species complex, including those that share the same amino acid sequence. In addition, relationships among species in the complex differ substantially in trees based on the nuclear *Ldh *and the mitochondrial *ND*5 (Figure 2) genes. For example, alleles from species in the tenebrosa group cluster with those from NA *D. pulicaria *in trees based on both *Ldh *loci. Moreover, *D. arenata *is the sister taxon to the other members of the complex in the *Ldh*B tree, although European *D. pulex *occupies this position in the *Ldh*A tree, which is consistent with the mtDNA phylogeny (Figure 2). Vergilino et al. [30] analyzed sequence variation in the nuclear *Rab*4 gene from lineages in the pulex species complex and also found that tenebrosa group alleles cluster with NA *D. pulicaria*. However, they found that *D. arenata *clusters with NA *D. pulex*, which is consistent with the mtDNA phylogeny. The only other phylogenetic analysis of this species complex based on nuclear genes is that of Omilian and Lynch [22], who analyzed variation at six nuclear protein-coding genes (one of which was *Rab*4) in *D. arenata*, NA *D. pulex *and NA *D. pulicaria*. They also found that the relationship between *D. arenata *alleles and alleles from the other two species varies across the six loci, which could be a consequence of hybridization and/or incomplete sorting of ancestral polymorphism [30].

The fact that *Daphnia *species within the pulex complex have hybridized in the past, and continue to do so, is well-known [38,41] and this undoubtedly contributes to the inconsistency between phylogenies based on mitochondrial and nuclear genes. Despite the lack of congruence across different nuclear loci, alleles from each species do tend to form monophyletic groups suggesting that gene pools in this species complex have been isolated long enough to diverge from one another at the nucleotide level. One explanation for this pattern is that the nuclear genomes of some species in this complex are mosaics of genes derived from multiple species generated by a complex history of recurrent introgressive hybridization followed by isolation and divergence [42]. Phylogenetic analysis of additional nuclear loci will be required to test this hypothesis.

### Hybridization and recombination

Ongoing hybridization between *D. pulex *and *D. pulicaria *should provide opportunities for recombination between the pulex and pulicaria allele groups at both *Ldh *loci, and some instances were observed in this study. Although the hybrids observed in nature appear to reproduce by obligate parthenogenesis, Heier & Dudycha [43] found that crosses between cyclically parthenogenetic *D. pulex *females and *D. pulicaria *males produce offspring that are themselves cyclically parthenogenetic, and are able to successfully backcross with both parental species and each other, with no apparent loss of sexual function. Indeed, we identified a group of 15 recombinant pulex *Ldh*A alleles with two tracts of sequence from pulicaria alleles that appear to have arisen in populations from the Canadian prairies, and are now geographically widespread. In addition, six of the nine isolates with "recombinant" *Ldh*A/*Ldh*B genotypes (Table 1) came from Saskatchewan and Manitoba, including the isolate that is a pulex *Ldh*A and pulicaria *Ldh*B homozygote. Although, the breeding system of these isolates is unknown, the pattern of *Ldh *variation we observed suggests that substantial introgressive hybridization may be occurring between *D. pulex *and *D. pulicaria *in this region, where bodies of water vary substantially in depth, surface area and the presence of fish, and thus provide a continuum of habitats between "ponds" and "lakes". This is also consistent with the occurrence of the *Ldh*-S allele at low frequency in lake populations from central and western U.S.A [11] and Oregon [20], and with the results of Omilian and Lynch [22] whose analysis indicated that gene flow between these two species is on the order of 1.7 gene migrants per generation.

### Sequence diversity in *D. pulex *and *D. pulicaria*

Nucleotide diversity at synonymous sites in pulex *Ldh*A (π_s _= 0.027) and *Ldh*B (π_s _= 0.031) alleles is similar to the average estimate of nuclear protein-coding genes in other invertebrates (π_s _= 0.027, [44]), and well within the range of values estimated by Omilian & Lynch [22] for six nuclear genes in NA *D. pulex *(mean π_s _= 0.019, range = 0.000 - 0.050) and NA *D. pulicaria *(π_s _= 0.015, range 0.000 to 0.037). In four of the six genes, estimates of π_s _are higher in *D. pulicaria *than in *D. pulex*. In contrast, synonymous nucleotide diversity in both pulicaria *Ldh*A (π_s _= 0.006) and *Ldh*B (π_s _= 0.007) alleles is substantially lower than that in *D. pulex *(π_s _= 0.027 for *Ldh*A, π_s _= 0.031 for *Ldh*B).

The neutrality tests are negative in all cases but only significant for pulicaria alleles (Table 3), which is consistent with the operation of purifying selection, a selective sweep and/or demographic expansion [45]. The latter two alternatives are also consistent with the lack of correlation between sequence divergence and geographic distance for the pulicaria alleles at both loci in the Mantel tests (Figure 7). However, Fay and Wu's H statistic is not significant for either gene, which does not support a selective sweep. Taken together, the lower level of genetic variation in both *Ldh *genes in *D. pulicaria*, and the lack of geographic structuring of this variation suggest a recent demographic expansion of lake populations over a very wide geographic area after the evolution of the fast allele at *Ldh*A. Indeed, isolates from SA *D. pulicaria *are heterozygous for a pulicaria-group allele at both *Ldh*A and *Ldh*B. The other alleles in these isolates form a distinct South American cluster. Recent expansion of *D. pulicaria *is also consistent with the results of Omilian and Lynch [22], which suggested that effective population size (N_e_) is much lower in *D. pulicaria *than *D. pulex*, and that the nuclear genomes of these two species diverged from a common ancestor on the order of 80,000 years ago.

*D. pulicaria *and *D. pulex *are relatively young species and retain the ability to hybridize, so it is not surprising that they share alleles at many allozyme loci [19,20]. Despite the occurrence of gene flow between them [22], they show divergence in physiology and life history traits [14,15] associated with adaptation to pond and lake habitats. Previous allozyme surveys have identified two loci in addition to *Ldh*, *Hexokinase *(*Hk*) and a *Peptidase *that are fixed or nearly so for alternate alleles in lake and pond populations [19]. It has been suggested that selection, directly on these loci or on loci to which they are closely linked, may be involved in this divergence. For example, Pfrender et al. [21] analyzed variation at 13 allozyme loci, 11 microsatellite loci and mtDNA in *Daphnia *populations from western Oregon. They identified two pond populations that are genetically distinct from the *D. arenata *that typically inhabit ponds in this region [20,21]. Instead, these two populations are very similar to Oregon lake *D. pulicaria *at all loci except *Ldh *and *Hk*, where they are fixed, or nearly so for the alleles *Ldh*-S and *Hk*-M, which invariably predominate in pond populations.

It is extremely difficult to detect positive selection on individual amino acid sites by applying standard statistical tests to sequences from one species or closely related species [46,47]. However, it has been shown that one or a few amino acid substitutions can cause adaptive changes in protein function [48-50]. LDH is known to be involved in response to hypoxia, and variation in levels and temporal patterns of dissolved oxygen are very different in ponds and lakes. For example, oxygen tension in shallow temporary ponds decreases while temperature increases throughout the spring and summer and *Daphnia *populations generally die by mid-summer due to anoxia or desiccation [14]. Conversely, deep lakes are generally stratified during the summer months and *Daphnia *take refuge in the cold hypolimnion to avoid fish predation [14,51]. Members of the pulex species complex tend to occupy temporary ponds, although species in the tenebrosa group also occur in permanent ponds and small lakes. However, *D. pulicaria *is the only species in the complex that has invaded large, stratified lakes. Moreover, the amino acid sequence of the slow LDHA protein is highly conserved despite substantial genetic variation at synonymous sites and introns indicating that this protein is generally under strong purifying selection. The slow and fast LDHA proteins only differ at two amino acid sites and neither change is involved in substrate or NAD binding. Even so, these changes could affect the catalytic properties of the enzyme, or other properties such as thermostability. For example, an amino acid substitution between LDHB alleles in the fish, *F. heteroclitus *increases thermostability of the proteins found in southern populations [8]. Overall, the strong association between *Ldh*A genotype and habitat, and different patterns of protein variation in the two *Ldh *loci, suggest that experimental studies to determine the adaptive significance of amino acid variation in LDHA are warranted.

## Conclusions

The two L-*Ldh *loci in the *Daphnia pulex *species complex show substantial variation at the nucleotide level, but the amino acid sequence of LDHA is much more conserved than that of LDHB suggesting that the intensity of purifying selection on *Ldh*A is much stronger. Patterns of nucleotide variation also indicate that the fast *Ldh*A allele arose very recently and spread rapidly over a very wide geographic area, possibly during a recent demographic expansion of *D. pulicaria *lake populations. Although lake-dwelling *D. pulicaria *hybridizes with the other lineages in the pulex complex, it remains distinct ecologically and genetically. This, coupled with the strong association between *Ldh*A genotype and habitat, suggests that experimental studies would be useful to determine if there is a link between the protein variants and differences in enzymatic function, which could then be evaluated for their effects on fitness to test the hypothesis that LDHA variation is adaptive.

## Authors' contributions

RF cloned the *Ldh *genes and collected the sequence data. TJC performed the diversity analyses and constructed the NJ trees. MEC constructed the Bayesian trees. DI performed the Mantel tests. TJC, MEC and DI conceived the study and participated in its design. All authors helped to draft the manuscript and approved the final version.

## Supplementary Material

Additional file 1 **Supplementary tables**. This PDF file contains 4 tables as follows. **1.1 - **Isolates from the *Daphnia pulex *species complex analyzed for this study. **1.2 - **List of *Ldh*A and *Ldh*B allele groups used in the nt diversity analysis of *D. pulex *and *D. pulicaria. ***1.3 - **Results of the recombination analysis among alleles of *Ldh*A in *D. pulicaria *and *D. pulex*. **1.4 - **Results of the recombination analysis among alleles of *Ldh*B in *D. pulicaria *and *D. pulex*. Click here for file

Additional file 2 ***Ldh*A nucleotide sequence alignment**. A sequential alignment of *Ldh*A sequences from members of the *Daphnia pulex *species complex in FastA and Nexus formats. Click here for file

Additional file 3 ***Ldh*B nucleotide sequence alignment**. A sequential alignment of *Ldh*B sequences from members of the *Daphnia pulex *species complex in FastA and Nexus formats. Click here for file

Additional file 4 **LDHA amino acid alignment**. Sequential alignment of LDHA sequences from members of the *Daphnia pulex *species complex in FastA and Nexus formats. Click here for file

Additional file 5 **LHDB amino acid alignment**. Sequential alignment of LDHB sequences from members of the *Daphnia pulex *species complex in FastA and Nexus formats. Click here for file

Additional file 6 **Intron phylogenies**. Bayesian phylogeny of **(a) ***Ldh*A and **(b) ***Ldh*B intron sequences from members of the *Daphnia pulex *species complex. Estimates of posterior probability larger than 50 are indicated on the nodes of the tree. The trees are rooted through the *Daphnia obtusa *sequence. Click here for file
