# Supplementary material for: Evolutionary factors affecting Lactate dehydrogenase A and B variation in the Daphnia pulex species complex
Source: BMC Evol Biol. 2011 Jul 18;11:212. doi: 10.1186/1471-2148-11-212 (PMC3231769; doi:10.1186/1471-2148-11-212)
Supplement: Additional file 1 — Supplementary tables. This PDF file contains 4 tables as follows. 1.1 - Isolates from the Daphnia pulex species complex analyzed for this study. 1.2 - List of LdhA and LdhB allele groups used in the nt diversity analysis of D. pulex and D. pulicaria. 1.3 - Results of the recombination analysis among alleles of LdhA in D. pulicaria and D. pulex. 1.4 - Results of the recombination analysis among alleles of LdhB in D. pulicaria and D. pulex. [file 1471-2148-11-212-S1.PDF]

Additional File 1. Table.1. Isolates from the *Daphnia pulex* species complex analyzed for this study.

1. C = D. pulicaria, X = D. pulex, CX = pulicaria/pulex hybrid, ARE = D. arenata, EPC = European D. pulicaria, EPX = European D. pulex, MEL = D. melanica, MID = D. middendorfiana, TEN = D. tenebrosa, SAPC = South American D. pulicaria, OBT = D. obtusa

2. Canadian provinces and USA states have 2-letter codes. Countries have 3-letter UN codes.

3. CP = cyclic parthenogenesis; OP = obligate parthenogenesis; U = unknown

4. *D. pulicaria* is subdivided into eastern (Pce) and western (PCw) lineages. The remaining codes refer to species as in column 1.

Species[1]	no.	Prov/St Country[2]	Habitat	LDHA genotype	Breeding System[3]	mtDNA[4]	Latitude	Longitude	Source Lab
C	1	ON	lake	FF	CP	PCe	44.88	-78.75	Ghabooli
C	2	ME	lake	FF	CP	PCe	44.84	-69.28	Lynch
C	3	MI	lake	FF	CP	PCw	42.65	-85.50	Lynch
C	4	PA	lake	FF	CP	PCw	41.13	-75.58	Lynch
C	5	SK	lake	FF	CP	PCw	52.13	-105.13	Crease
C	6	WY	lake	FF	CP	PCw	44.55	-110.38	Crease
C	7	IL	lake	FF	CP	PX	40.14	-87.74	Caceres
C	8	ID	lake	FF	CP	PX	42.35	-112.44	Lynch
C	9	IL	lake	FF	CP	PX	40.13	-87.73	Caceres
C	10	IL	lake	FF	CP	PX	40.15	-87.74	Caceres
C	11	WA	lake	FF	CP	PX	47.61	-122.24	Hebert
C	12	QC	pond	FF	OP	PCw	55.28	-77.75	Dufresne
C	13	SK	prairie pothole	FF	U	PCw	49.42	-105.14	Crease
C	14	SK	prairie pothole	FF	U	PCw	49.42	-105.14	Crease
C	15	MB	prairie pothole	FF	U	PX	49.16	-96.30	Crease
C	16	MB	prairie pothole	FF	U	PX	50.04	-100.32	Crease
C	17	SK	prairie pothole	FF	U	PX	50.92	-102.40	Crease
CX	1	ISL	pond	SF	OP	PCe	64.09	-21.58	Weider
CX	2	QC	pond	SF	OP	PCe	55.28	-77.75	Dufresne
CX	3	MI	pond	SF	OP	PX	42.21	-83.70	Crease
CX	4	ME	pond	SF	OP	PX	42.99	-70.61	Herrish/Hairston
CX	5	NB	pond	SF	OP	PX	47.00	-67.47	Lynch
CX	6	NB	pond	SF	OP	PX	47.00	-67.47	Lynch
CX	7	IN	pond	SF	OP	PX	39.12	-87.31	Dudycha
CX	8	ON	pond	SF	OP	PX	42.12	-82.98	Cristescu
CX	9	ON	pond	SF	OP	PX	42.16	-83.02	Cristescu
CX	10	ME	pond	SF	OP	PX	44.86	-69.80	Lynch
CX	11	MI	pond	SF	OP	PX	42.17	-83.72	Innes
CX	12	NB	pond	SF	OP	PX	47.62	-65.67	Lynch
CX	13	NT	pond	SF	OP	PX	69.43	-133.02	Crease
CX	14	ON	pond	SF	U	PX	49.82	-93.23	Crease
CX	15	MB	pond	SF	U	PX	49.64	-98.24	Crease
CX	16	MB	pond	SF	U	PX	49.64	-98.24	Crease
CX	17	SK	pond	SF	U	PX	50.07	-105.53	Crease
CX	18	SK	pond	SF	U	PX	51.27	-103.72	Crease
X	1	MI	pond	SS	CP	PX	42.19	-83.58	Crease
X	2	IL	pond	SS	CP	PX	40.12	-88.20	Caceres
X	3	WI	pond	SS	CP	PX	46.67	-90.92	Lynch
X	4	IL	pond	SS	CP	PX	40.06	-87.92	Crease

Species[1]	no.	Prov/St Country [2]	Habitat	LDHA genotype	Breeding System [3]	mtDNA [4]	Latitude	Longitude	Source Lab
X	5	ON	pond	SS	CP	PX	42.17	-83.03	Innes
X	6	MN	pond	SS	CP	PX	44.97	-93.32	Lynch
X	7	ON	pond	SS	CP	PX	42.25	-83.02	Crease
X	8	IL	pond	SS	CP	PX	40.14	-87.74	Lynch
X	9	ON	pond	SS	CP	PX	42.67	-80.40	Crease
X	10	IN	pond	SS	CP	PX	39.90	-84.93	Lynch
X	11	IN	pond	SS	CP	PX	40.22	-87.33	Crease
X	12	MI	pond	SS	CP	PX	42.75	-85.35	Lynch
X	13	MI	pond	SS	CP	PX	42.2	-83.72	Lynch
X	14	QC	pond	SS	OP	PX	47.44	-72.78	Crease
X	15	QC	pond	SS	OP	PX	46.37	-71.19	Crease
X	16	MI	pond	SS	OP	PX	42.33	-83.66	Crease
X	17	ON	pond	SS	OP	PX	43.54	-80.21	Crease
X	18	NY	pond	SS	OP	PX	42.98	-78.77	Lynch
X	19	MI	pond	SS	OP	PX	46.06	-86.79	Lynch
X	20	WI	pond	SS	OP	PX	45.66	-88.10	Lynch
X	21	OR	pond	SS	OP	PX	45.12	-123.03	Pfrender
X	22	QC	pond	SS	OP	PX	48.13	-69.17	Lynch
X	23	QC	pond	SS	OP	PX	46.03	-73.45	Lynch
X	24	MI	pond	SS	OP	PX	46.01	-86.66	Lynch
X	25	MN	pond	SS	OP	PX	47.29	-92.49	Lynch
X	26	ME	pond	SS	OP	PX	46.50	-68.22	Lynch
X	27	NY	pond	SS	OP	PX	42.47	-76.37	Lynch
X	28	ON	pond	SS	OP	PX	42.33	-81.84	Lynch
X	29	ME	pond	SS	OP	PX	44.63	-69.23	Lynch
X	30	OH	pond	SS	OP	PX	41.34	-81.16	Lynch
X	31	ON	pond	SS	U	PX	49.82	-93.23	Crease
X	32	SK	pond	SS	U	PX	50.07	-105.53	Crease
X	33	SK	pond	SS	U	PX	49.85	-105.04	Crease
X	34	SK	pond	SS	U	PX	49.85	-105.04	Crease
X	35	IL	pond	SS	U	PX	41.69	-89.27	Geedey
ARE	1	OR	pond	SS	CP	AR	43.94	-123.05	Lynch
ARE	2	OR	pond	SS	CP	AR	43.83	-124.12	Lynch
ARE	3	OR	pond	SS	CP	AR	44.03	-123.15	Lynch
MEL		OR	pond	FF	CP	ME	43.97	-124.11	Lynch
MID	1	MB	pond	SF	OP	MI	58.46	-93.51	Dufresne
MID	2	NUN	pond	SS	OP	MI	72.68	-77.94	Weider
EPC	1	CZE	lake	SS	CP	EC	50.08	14.48	Dufresne
EPC	2	ESP	lake	SS	CP	EC	42.63	1.00	Dufresne
EPC	3	CZE	lake	SS	CP	EC	50.18	12.88	Adam
TEN		NT	pond	SS	OP	TE	70.29	-127.50	Weider
SAPC	1	BOL	lake	SF	OP	SA-C	-17.04	-66.61	Mergeay
SAPC	2	PER	lake	SF	U	SA-C	-15.70	-69.5	Adamowicz
SAPC	3	BOL	lake	SF	U	SA-C	-15.80	-69.38	Adamowicz
EPX		CZE	pond	SS	CP	EX	48.88	14.63	Adam
OBT		IL	pond	SS	CP	OB	40.13	-88.15	Lynch

Additional File 1. Table 2. List of LdhA and LdhB allele groups used in the nt diversity analysis of *D. pulex* and *D. pulicaria*

NOTE Alleles from hybrids involving species other than North American *D. pulex* or *D. pulicaria* were not included in the diversity analyses.

LDHA	group X
X	01A_MI
X	01B_MI
X	02A_IL
X	02B_IL
X	03A_WI
X	03B_WI
X	04A_IL
X	04B_IL
X	05_ON
X	06A_MN
X	06B_MN
X	07_ON
X	08A_IL
X	08B_IL
X	09_ON
X	10_IN
X	11A_IN
X	11B_IN
X	12A_MI
X	12B_MI
X	13A_MI
X	13B_MI
X	014A_QC
X	014B_QC
X	015A_QC
X	15B_QC
X	16A_MI
X	16B_MI
X	17_ON
X	18A_NY
X	18B_NY
X	19A_MI
X	19B_MI
X	20A_WI
X	20B_WI
X	21_OR
X	22_QC
X	23A_QC
X	23B_QC
X	24_MI
X	25A_MN
X	25B_MN
X	26_ME
X	27_NY
X	28A_ON
X	28B_ON
X	29A_ME
X	29B_ME
X	30A_OH

LDHA	group C
C	01_ON
C	02_ME
C	03_MI
C	04_PA
C	05A_SK
C	05B_SK
C	06A_WY
C	06B_WY
C	07_IL
C	08A_ID
C	08B_ID
C	09_IL
C	10_IL
C	11_WA
C	12A_QC
C	12B_QC
C	13_SK
C	14A_SK
C	14B_SK
C	15A_MB
C	15B_MB
C	16A_MB
C	16B_MB
C	17_SK
CX	01A_ICE
CX	02A_QC
CX	03A_MI
CX	04A_ME
CX	05A_NB
CX	06A_NB
CX	07B_IN
CX	08A_ON
CX	09A_ON
CX	10A_ME
CX	11A_MI
CX	12A_NB
CX	13A_NT
CX	14A_ON
CX	15A_MB
CX	16A_MB
CX	17A_SK
CX	18A_SK

N=42

LDHB	group X
X	01A_MI
X	01B_MI
X	02A_IL
X	02B_IL
X	03A_WI
X	03B_WI
X	04A_IL
X	04B_IL
X	05A_ON
X	05B_ON
X	06A_MN
X	06B_MN
X	07A_ON
X	07B_ON
X	08_IL
X	09A_ON
X	09B_ON
X	10A_IN
X	10B_IN
X	11_IN
X	12A_MI
X	12B_MI
X	13A_MI
X	13B_MI
X	14A_QC
X	14B_QC
X	15A_QC
X	15B_QC
X	16A_MI
X	16B_MI
X	17_ON
X	18A_NY
X	18B_NY
X	19A_MI
X	19B_MI
X	20_WI
X	21_OR
X	22_QC
X	23A_QC
X	23B_QC
X	24A_MI
X	24B_MI
X	25A_MN
X	25B_MN
X	26_ME
X	27A_NY
X	27B_NY
X	28A_ON
X	28B_ON

LDHB	group C
C	01A_ON
C	01B_ON
C	02A_ME
C	02B_ME
C	03A_MI
C	03B_MI
C	04A_PA
C	04B_PA
C	05A_SK
C	05B_SK
C	06A_WY
C	06B_WY
C	07A_IL
C	07B_IL
C	08A_ID
C	08B_ID
C	09A_IL
C	09B_IL
C	10A_IL
C	10B_IL
C	11A_WA
C	11B_WA
C	12A_QC
C	12B_QC
C	13A_SK
C	13B_SK
C	14A_SK
C	14B_SK
C	15A_MB
C	15B_MB
C	16A_MB
C	16B_MB
C	17A_SK
C	17B_SK
CX	01A_ICE
CX	02B_QC
CX	03B_MI
CX	04A_ME
CX	06B_NB
CX	07A_IN
CX	08A_ON
CX	09A_ON
CX	10A_ME
CX	11B_MI
CX	12A_NB
CX	13B_NT
CX	14B_ON
CX	15A_MB
CX	16A_MB

X	30B_OH
X	31_ON
X	32A_SK
X	32B_SK
X	33A_SK
X	33B_SK
X	34A_SK
X	34B_SK
X	35A_IL
X	35B_IL
CX	02B_QC
CX	04B_ME
CX	05B_NB
CX	06B_NB
CX	07A_IN
CX	08B_ON
CX	09B_ON
CX	10B_ME
CX	11B_MI
CX	12B_NB
CX	13B_NT
CX	14B_ON
CX	15B_MB
CX	16B_MB
CX	17B_SK
CX	18B_SK

N=75

X	29A_ME
X	29B_ME
X	30B_OH
X	31A_ON
X	31B_ON
X	32A_SK
X	33A_SK
X	35B_IL
CX	02A_QC
CX	03A_MI
CX	04B_ME
CX	05_NB
CX	06A_NB
CX	07B_IN
CX	08B_ON
CX	09B_ON
CX	10B_ME
CX	11A_MI
CX	12B_NB
CX	13A_NT
CX	14A_ON
CX	15B_MB

N=71

CX	16B_MB
CX	17A_SK
CX	17B_SK
CX	18A_SK
CX	18B_SK
X	30A_OH
X	32B_SK
X	33B_SK
X	34_SK
X	35A_IL

N=59

NOTES

LDHA-CX-03B_MI is an MI allele and is omitted
LDHA-CX-01B_ICE is an EC allele and is omitted
LDHB-CX-01B_ICE is an EC allele and is omitted

Additional File 1. Table 3. Results of the recombination analysis among alleles of *Ldh* A in *D. pulicaria* and *D. pulex* .

Population 1: LDHA_pulex **Number of sequences: 75**
Population 2: LDHA_pulicaria **Number of sequences: 42**

**Number of Gene Conversion tracts identified: 30**			**group**
Line: LDHA-CX-02b_QC (population 1). Tract located between sites: 812-839			
Nucleotide distance (excluding gaps): 28			X
Line: LDHA-CX-02b_QC (population 1). Tract located between sites: 1161-1280			
Nucleotide distance (excluding gaps): 74			X
Line: LDHA-CX-07a_IN (population 1). Tract located between sites: 812-839			
Nucleotide distance (excluding gaps): 28			C
Line: LDHA-CX-07a_IN (population 1). Tract located between sites: 1161-1280			
Nucleotide distance (excluding gaps): 74			X
Line: LDHA-CX-13b_NT (population 1). Tract located between sites: 812-839			
Nucleotide distance (excluding gaps): 28			X
Line: LDHA-CX-13b_NT (population 1). Tract located between sites: 1161-1280			
Nucleotide distance (excluding gaps): 74			X
Line: LDHA-CX-14b_ON (population 1). Tract located between sites: 1161-1280			
Nucleotide distance (excluding gaps): 74			X
Line: LDHA-CX-15b_MB (population 1). Tract located between sites: 812-839			
Nucleotide distance (excluding gaps): 28			X
Line: LDHA-CX-15b_MB (population 1). Tract located between sites: 1161-1280			
Nucleotide distance (excluding gaps): 74			X
Line: LDHA-CX-16b_MB (population 1). Tract located between sites: 812-839			
Nucleotide distance (excluding gaps): 28			X
Line: LDHA-CX-16b_MB (population 1). Tract located between sites: 1161-1280			
Nucleotide distance (excluding gaps): 74			X
Line: LDHA-CX-17b_SK (population 1). Tract located between sites: 812-839			
Nucleotide distance (excluding gaps): 28			X
Line: LDHA-CX-17b_SK (population 1). Tract located between sites: 1161-1280			
Nucleotide distance (excluding gaps): 74			X
Line: LDHA-CX-18b_SK (population 1). Tract located between sites: 812-839			
Nucleotide distance (excluding gaps): 28			X
Line: LDHA-CX-18b_SK (population 1). Tract located between sites: 1161-1280			
Nucleotide distance (excluding gaps): 74			X
Line: LDHA-X-06b_MN (population 1). Tract located between sites: 812-839			
Nucleotide distance (excluding gaps): 28			X
Line: LDHA-X-06b_MN (population 1). Tract located between sites: 1161-1280			
Nucleotide distance (excluding gaps): 74			X
Line: LDHA-X-02a_SK (population 1). Tract located between sites: 812-839			
Nucleotide distance (excluding gaps): 28			X
Line: LDHA-X-32b_SK (population 1). Tract located between sites: 812-839			
Nucleotide distance (excluding gaps): 28			X
Line: LDHA-X-32b_SK (population 1). Tract located between sites: 1161-1280			
Nucleotide distance (excluding gaps): 74			X
Line: LDHA-X-33a_SK (population 1). Tract located between sites: 812-839			
Nucleotide distance (excluding gaps): 28			X
Line: LDHA-X-33a_SK (population 1). Tract located between sites: 1161-1280			
Nucleotide distance (excluding gaps): 74			X
Line: LDHA-X-33b_SK (population 1). Tract located between sites: 812-839			
Nucleotide distance (excluding gaps): 28			X
Line: LDHA-X-33b_SK (population 1). Tract located between sites: 1161-1280			
Nucleotide distance (excluding gaps): 74			X
Line: LDHA-X-34a_SK (population 1). Tract located between sites: 812-839			
Nucleotide distance (excluding gaps): 28			X
Line: LDHA-X-34a_SK (population 1). Tract located between sites: 1161-1280			
Nucleotide distance (excluding gaps): 74			X
Line: LDHA-X-34b_SK (population 1). Tract located between sites: 812-839			
Nucleotide distance (excluding gaps): 28			X
Line: LDHA-X-34b_SK (population 1). Tract located between sites: 1161-1280			
Nucleotide distance (excluding gaps): 74			X
Line: LDHA-X-35b_IL (population 1). Tract located between sites: 812-839			
Nucleotide distance (excluding gaps): 28			X
Line: LDHA-CX-07b_IN (population 2). Tract located between sites: 18-145			
Nucleotide distance (excluding gaps): 127			C

Additional File 1. Table 4. Results of the recombination analysis among alleles of *Ldh* B in *D. pulicaria* and *D. pulex*.

Population 1: LDHB_pulex **Number of sequences: 71**
Population 2: LDHB_pulicaria **Number of sequences: 59**

Number of Gene Conversion tracts identified: 15

			**group**
Line: 'LDHB-CX-03b_MI' (population 1). Tract located between sites: 1429-1506			
Nucleotide distance (excluding gaps): 76			C
Line: 'LDHB-CX-02a_QC' (population 2). Tract located between sites: 772-781			
Nucleotide distance (excluding gaps): 10			X
Line: 'LDHB-CX-03a_MI' (population 2). Tract located between sites: 772-781			
Nucleotide distance (excluding gaps): 10			X
Line: 'LDHB-CX-04b_ME' (population 2). Tract located between sites: 467-468			
Nucleotide distance (excluding gaps): 2			X
Line: 'LDHB-CX-04b_ME' (population 2). Tract located between sites: 772-781			
Nucleotide distance (excluding gaps): 10			X
Line: 'LDHB-CX-13a_NT' (population 2). Tract located between sites: 772-781			
Nucleotide distance (excluding gaps): 10			X
Line: 'LDHB-X-04a_IL' (population 2). Tract located between sites: 772-781			
Nucleotide distance (excluding gaps): 10			X
Line: 'LDHB-X-05b_ON' (population 2). Tract located between sites: 772-781			
Nucleotide distance (excluding gaps): 10			X
Line: 'LDHB-X-27a_NY' (population 2). Tract located between sites: 772-781			
Nucleotide distance (excluding gaps): 10			X
Line: 'LDHB-X-29b_ME' (population 2). Tract located between sites: 772-781			
Nucleotide distance (excluding gaps): 10			X
Line: 'LDHB-X-30b_OH' (population 2). Tract located between sites: 1429-1506			
Nucleotide distance (excluding gaps): 76			X
Line: 'LDHB-X-32a_SK' (population 2). Tract located between sites: 772-1296			
Nucleotide distance (excluding gaps): 501			X
Line: 'LDHB-X-33a_SK' (population 2). Tract located between sites: 467-468			
Nucleotide distance (excluding gaps): 2			X
Line: 'LDHB-X-35b_IL' (population 2). Tract located between sites: 467-468			
Nucleotide distance (excluding gaps): 2			X
Line: 'LDHB-X-35b_IL' (population 2). Tract located between sites: 772-781			
Nucleotide distance (excluding gaps): 10			X
