# Supplementary material for: Evolutionary factors affecting Lactate dehydrogenase A and B variation in the Daphnia pulex species complex
Source: BMC Evol Biol. 2011 Jul 18;11:212. doi: 10.1186/1471-2148-11-212 (PMC3231769; doi:10.1186/1471-2148-11-212)
Supplement: Additional file 6 — Intron phylogenies. Bayesian phylogeny of (a) LdhA and (b) LdhB intron sequences from members of the Daphnia pulex species complex. Estimates of posterior probability larger than 50 are indicated on the nodes of the tree. The trees are rooted through the Daphnia obtusa sequence. [file 1471-2148-11-212-S6.PDF]

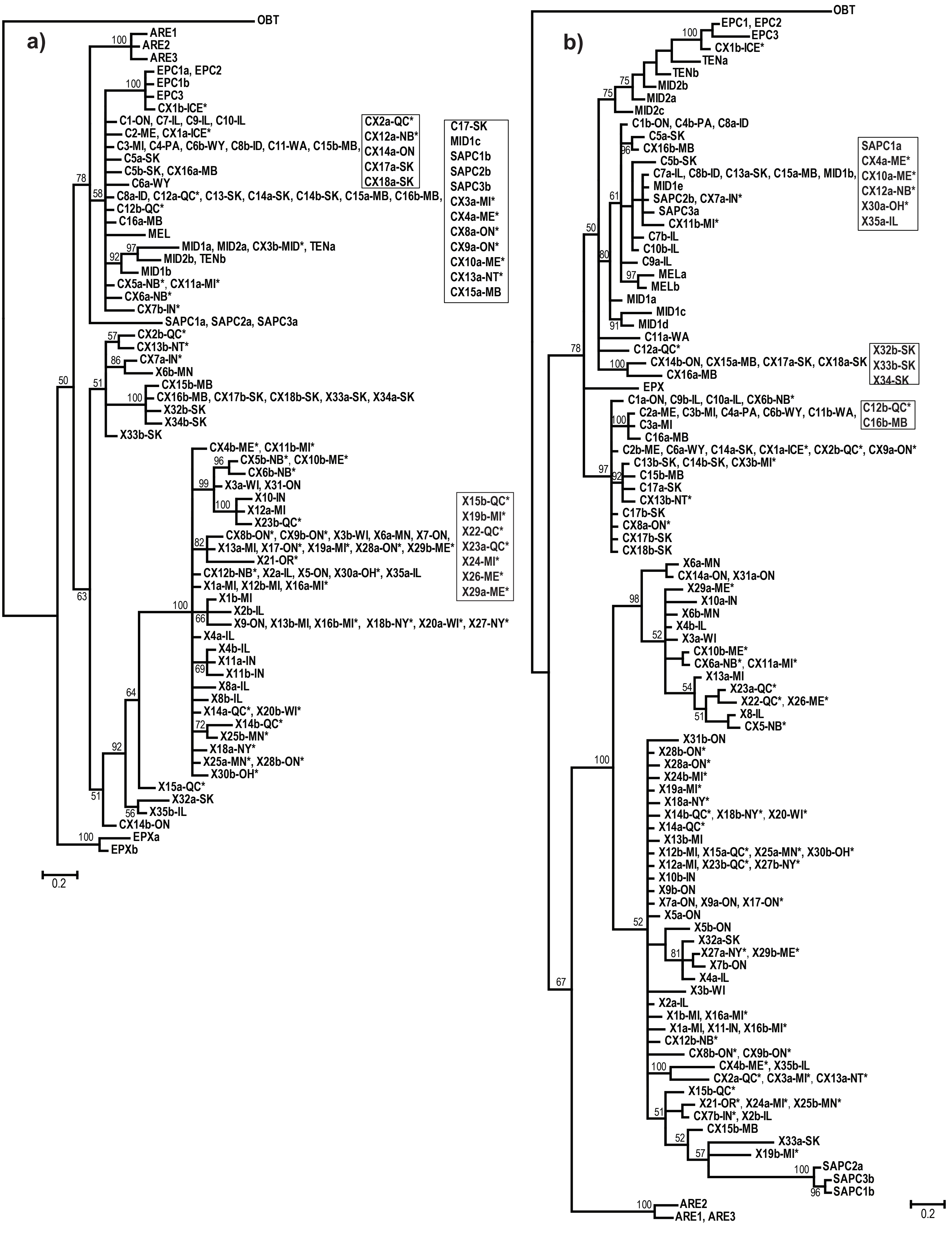

a)
OBT
ARE1
ARE2
ARE3
EPC1a, EPC2
EPC1b
EPC3
CX1b-ICE*
C1-ON, C7-IL, C9-IL, C10-IL
C2-ME, CX1a-ICE*
C3-MI, C4-PA, C6b-WY, C8b-ID, C11-WA, C15b-MB,
C5a-SK
C5b-SK, CX16a-MB
C6a-WY
C8a-ID, C12a-QC*, C13-SK, C14a-SK, C14b-SK, C15a-MB, C16b-MB,
C12b-QC*
C16a-MB
MEL
MID1a, MID2a, CX3b-MID*, TENa
MID2b, TENb
MID1b
CX5a-NB*, CX11a-MI*
CX6a-NB*
CX7b-IN*
SAPC1a, SAPC2a, SAPC3a
CX2a-QC*
CX12a-NB*
CX14a-ON
CX17a-SK
CX18a-SK
C17-SK
MID1c
SAPC1b
SAPC2b
SAPC3b
CX3a-MI*
CX4a-ME*
CX8a-ON*
CX9a-ON*
CX10a-ME*
CX13a-NT*
CX15a-MB
CX2b-QC*
CX13b-NT*
CX7a-IN*
X6b-MN
CX15b-MB
CX16b-MB, CX17b-SK, CX18b-SK, X33a-SK, X34a-SK
X32b-SK
X34b-SK
X33b-SK
CX4b-ME*, CX11b-MI*
CX5b-NB*, CX10b-ME*
CX6b-NB*
X3a-WI, X31-ON
X10-IN
X12a-MI
X23b-QC*
CX8b-ON*, CX9b-ON*, X3b-WI, X6a-MN, X7-ON,
X13a-MI, X17-ON*, X19a-MI*, X28a-ON*, X29b-ME*
X21-OR*
CX12b-NB*, X2a-IL, X5-ON, X30a-OH*, X35a-IL
X1a-MI, X12b-MI, X16a-MI*
X1b-MI
X2b-IL
X9-ON, X13b-MI, X16b-MI*, X18b-NY*, X20a-WI*, X27-NY*
X4a-IL
X4b-IL
X11a-IN
X11b-IN
X8a-IL
X8b-IL
X14a-QC*, X20b-WI*
X14b-QC*
X25b-MN*
X18a-NY*
X25a-MN*, X28b-ON*
X30b-OH*
X15b-QC*
X19b-MI*
X22-QC*
X23a-QC*
X24-MI*
X26-ME*
X29a-ME*
X15a-QC*
X32a-SK
X35b-IL
CX14b-ON
EPXa
EPXb
0.2
b)
OBT
EPC1, EPC2
EPC3
CX1b-ICE*
TENa
TENb
MID2b
MID2a
MID2c
C1b-ON, C4b-PA, C8a-ID
C5a-SK
CX16b-MB
C5b-SK
C7a-IL, C8b-ID, C13a-SK, C15a-MB, MID1b,
MID1e
SAPC2b, CX7a-IN*
SAPC3a
CX11b-MI*
C7b-IL
C10b-IL
C9a-IL
MELa
MELb
MID1a
MID1c
MID1d
C11a-WA
C12a-QC*
CX14b-ON, CX15a-MB, CX17a-SK, CX18a-SK
CX16a-MB
SAPC1a
CX4a-ME*
CX10a-ME*
CX12a-NB*
X30a-OH*
X35a-IL
X32b-SK
X33b-SK
X34-SK
EPX
C1a-ON, C9b-IL, C10a-IL, CX6b-NB*
C2a-ME, C3b-MI, C4a-PA, C6b-WY, C11b-WA,
C3a-MI
C16a-MB
C12b-QC*
C16b-MB
C2b-ME, C6a-WY, C14a-SK, CX1a-ICE*, CX2b-QC*, CX9a-ON*
C13b-SK, C14b-SK, CX3b-MI*
C15b-MB
C17a-SK
CX13b-NT*
C17b-SK
CX8a-ON*
CX17b-SK
CX18b-SK
X6a-MN
CX14a-ON, X31a-ON
X29a-ME*
X10a-IN
X6b-MN
X4b-IL
X3a-WI
CX10b-ME*
CX6a-NB*, CX11a-MI*
X13a-MI
X23a-QC*
X22-QC*, X26-ME*
X8-IL
CX5-NB*
X31b-ON
X28b-ON*
X28a-ON*
X24b-MI*
X19a-MI*
X18a-NY*
X14b-QC*, X18b-NY*, X20-WI*
X14a-QC*
X13b-MI
X12b-MI, X15a-QC*, X25a-MN*, X30b-OH*
X12a-MI, X23b-QC*, X27b-NY*
X10b-IN
X9b-ON
X7a-ON, X9a-ON, X17-ON*
X5a-ON
X5b-ON
X32a-SK
X27a-NY*, X29b-ME*
X7b-ON
X4a-IL
X3b-WI
X2a-IL
X1b-MI, X16a-MI*
X1a-MI, X11-IN, X16b-MI*
CX12b-NB*
CX8b-ON*, CX9b-ON*
CX4b-ME*, X35b-IL
CX2a-QC*, CX3a-MI*, CX13a-NT*
X15b-QC*
X21-OR*, X24a-MI*, X25b-MN*
CX7b-IN*, X2b-IL
CX15b-MB
X33a-SK
X19b-MI*
SAPC2a
SAPC3b
SAPC1b
ARE2
ARE1, ARE3
0.2
